# Emerging Applications of Biomedical Science in Pandemic Prevention and Control: A Review

**DOI:** 10.7759/cureus.44075

**Published:** 2023-08-24

**Authors:** Induni N Weerarathna, Anurag Luharia, Suhas Tivaskar, Francis A Nankong, David Raymond

**Affiliations:** 1 Biomedical Sciences, Datta Meghe Institute of Higher Education and Research, Wardha, IND; 2 Medical Physics, Radiology, Radiotherapy, Nuclear Medicine, Radiobiology, and Radiation Safety, Datta Meghe Institute of Higher Education and Research, Wardha, IND; 3 Radiology, Datta Meghe Institute of Higher Education and Research, Wardha, IND; 4 Science and Technology, Datta Meghe Institute of Higher Education and Research, Wardha, IND; 5 Computer Science and Medical Engineering, Datta Meghe Institute of Higher Education and Research, Wardha, IND

**Keywords:** sars-cov-2, coronavirus disease, pandemic prevention and control, biomedical science, nanotechnology, equipment, therapies, vaccines, diagnostics

## Abstract

The COVID-19 pandemic has made it abundantly clear how crucial biomedical science is to pandemic control and prevention on a global scale. The importance of biomedical science in the fight against pandemics has increased with the appearance of new, deadly infectious diseases. Biomedical science and engineering have been presented as possible areas for combating the severe acute respiratory syndrome coronavirus 2 (SARS-CoV-2) due to the unique challenges raised by the pandemic, as reported by epidemiologists, immunologists, and doctors, including the survival, symptoms, protein surface composition, and infection mechanisms of COVID-19. These multidisciplinary engineering concepts are applied to design and develop prevention methods, diagnostics, monitoring, and therapies. An infectious disease outbreak that has spread over a sizable region, such as several continents or the entire world, and is affecting a sizable number of people is referred to as a "pandemic. While current knowledge about the SARS-CoV-2 virus is still limited, various (old and new) biomedical approaches have been developed and tested.

Here, we review the emerging applications of biomedical science in pandemic prevention and control, including rapid diagnosis tests, the development of vaccines, antiviral therapies, artificial intelligence, genome sequencing, and personal protective equipment. Biomedical science and nanotechnology are two fields that have the potential to combine to develop emerging applications for combating pandemics. In this review, we also discuss the intersection of biomedical science and nanotechnology in pandemic prevention and control.

## Introduction and background

Biomedical science is an interdisciplinary field that includes various branches of science such as biology, chemistry, physics, and engineering. It is essential for comprehending illnesses, creating cures, and stopping pandemics from spreading. The most recent COVID-19 pandemic has brought to light the value of biomedical science in pandemic control and prevention. Biomedical scientists in the biomedical field are examining the virus's modes of airborne and surface contamination spread. The development of public health policies to stop the virus's spread is made easier with the use of this information [1].

From a biological perspective, the current processes of rapid change in natural variables produce ideal circumstances for the appearance of new and mutational existing populations of biological structures at various levels, such as bacteria, viruses, and microorganisms. The origin and spread of several infectious diseases, pandemics, and the active transmission of dangerous bacteria and viruses are all influenced by this. Such catastrophic disasters have affected humanity quite frequently during the last few centuries, killing millions of people. Diseases including smallpox, plague, cholera, typhoid, flu, tuberculosis (TB), malaria, leprosy, human immunodeficiency virus (HIV) infection, and coronary virus infection are among these catastrophes. Of the seven recorded cholera pandemics, the most recent, which spread across Asia in the 1960s, took millions of lives. Of the seven recorded cholera pandemics, the most recent, which spread across Asia in the 1960s, took millions of lives. Typhoid entered Europe at the start of the 19th century with the millionth sacrifice. Malaria is common in tropical and subtropical areas, encompassing both America and Asia, as well as Africa. Each year, there are up to 500 million cases of malaria reported, of which up to three million result in mortality. Around the world, eight million people contract TB each year, and two million pass away from it. About 100 million people perished from tuberculosis, influenza, and other diseases in the 20th century [2].

Using the advancements of contemporary science and technology in their various fields, including microbiology, medicine, pharmacology, and several natural sciences, it was always possible for people to find ways to prevent and effectively treat these severe infectious diseases. All of these scientific breakthroughs, however, would not have been feasible without the application of evolving techniques and tools for observing biological and technological processes. These include cutting-edge methods for observing and influencing natural processes, methods for conducting analytical research and actively influencing it, tools and techniques for processing research findings, as well as technological advancements that aid in the detection and treatment of infectious diseases that affect large populations [1].

In terms of the medical side, a scientific study to identify the most efficient ways of medical diagnosis and screening for a pandemic of this sort, established based on previously identified primary signs and symptoms characteristic of this infection, is particularly pertinent at this point. The methods of geographically distributed, remote, mobile diagnostics, which are distinctive of telemedicine diagnostic complexes and telemedicine screening systems, are also particularly effective when taking into account the characteristics of viral pandemics for rapid and active spread among the population over large areas [1].

Currently, telemedicine allows for the use of all currently accepted medical examination and analysis techniques, including anamnesis, an objective assessment of the patient's physical condition, analysis of the results of laboratory tests on blood and other bodily secretions, X-ray studies, graphic methods, endoscopy, biopsy, and others. These are clinical research techniques, which include laboratory investigations, pathogen analysis, and the identification of antibodies to this virus in the patient's blood. As a result, biomedical scientists have been able to find more applications to prevent and control pandemic situations [1].

One of the emerging applications of biomedical science in pandemic prevention is the development of diagnostic tools. Diagnostic tools are crucial for identifying those who have viruses like severe acute respiratory syndrome coronavirus 2 (SARS-CoV-2), which causes COVID-19. Biomedical scientists are developing various diagnostic tools, including rapid antigen tests, polymerase chain reaction (PCR) tests, and serological tests, to detect COVID-19 in individuals. These tests help to isolate infected individuals and prevent the further spread of the virus. Another application of biomedical science in pandemic prevention is the development of therapeutics. Biomedical scientists are developing drugs and treatments to combat the COVID-19 virus. These include antiviral drugs, monoclonal antibodies, and convalescent plasma. They are also developing vaccines to prevent COVID-19 infection [2].

Biomedical science is playing a critical role in understanding the transmission of COVID-19. Biomedical scientists are studying the virus's modes of transmission, including airborne transmission and surface contamination. This information helps to develop public health guidelines to prevent the spread of the virus. Biomedical science is also developing technologies to monitor the spread of the virus. For example, researchers are using wastewater surveillance to detect the presence of SARS-CoV-2 in communities. This technology helps to identify outbreaks before they become widespread, allowing public health officials to respond quickly and contain the spread of the virus [3].

Biomedical scientists are going to make personal protective equipment (PPE) with the help of biotechnology, including nanotechnology-based coatings, biosensors, wearable technology, and 3D printing. These technologies provide enhanced protection against viral transmission and reduce the risk of infection for frontline workers and the general population. Additionally, biomedical science has aided in the creation of vaccines, which have been essential in halting the virus's spread. The vaccines have been developed and tested at an unprecedented pace, thanks to advancements in biomedical science. This has been a significant achievement, and it provides hope for the control of other infectious diseases in the future [4].

## Review

Search methodology

A thorough search of academic databases like PubMed, Web of Science, and Scopus was used as part of the methodology for this review article to find pertinent studies, reviews, and reports on the newly developed uses of biomedical science in pandemic prevention and control. Articles released between 2010 and the present were included in the search. It used precise keywords like "diagnostics", "prevention", "vaccines", "therapies", "equipment", and "nanotechnology".

Based on the inclusion and exclusion criteria, pertinent articles, reviews, and reports that address the research question are chosen. The inclusion criteria required that the articles be published in the English language, discuss advances in biomedical science that can be used during pandemics worldwide, discuss the effects of the COVID-19 pandemic on vaccination programs, discuss both observational and interventional studies, and be published between 2010 and the present.

Emerging applications of biomedical science

Rapid Diagnosis Tests (RDTs)

Rapid diagnostic tests (RDTs) for pandemic prevention and control have been developed and implemented in large part. Thanks to the contributions of biomedical science, scientists are working to develop new RDTs for the detection of emerging infectious diseases, such as COVID-19. They are also developing RDTs that are more accurate, sensitive, and specific than existing tests. For example, rapid diagnostic tests called COVID-19 antigen tests identify specific proteins from the COVID-19-causing SARS-CoV-2 virus. Since the beginning of the pandemic, several fresh COVID-19 antigen tests have been created, including the Sofia SARS Antigen Fluorescent Immunoassay (FIA) and BinaxNOW COVID-19 Ag Card. The gene editing technique is used by Clustered Regularly Interspaced Short Palindromic Repeats (CRISPR)-based RDTs to find viral DNA or ribonucleic acid (RNA) sequences in samples. These tests can be extremely sensitive and specific [5].

Examples of CRISPR-based RDTs for COVID-19 include the Sherlock CRISPR SARS-CoV-2 Kit from Sherlock Biosciences and the SARS-CoV-2 DETECTR assay from Mammoth Biosciences. To find a specific pathogen or antibody, fluorescent dyes are used in fluorescence-based RDTs. These examinations may be quite sensitive and precise. The LumiraDx SARS-CoV-2 Ag Test and the Gene Xpert Flu/RSV XC are two examples of RDTs that use fluorescence as an indicator. Isothermal amplification is when RDTs amplify specific RNA or DNA sequences to detect the presence of a pathogen. The use of expensive equipment is not necessary because these tests can be carried out at a constant temperature. The RT-LAMP COVID-19 Kit and the ID NOW COVID-19 test are two examples of isothermal amplification RDTs. Multiplexed RDTs can detect multiple pathogens or antibodies in a single test. These tests can be highly efficient and useful for surveillance and mass testing. Examples of multiplexed RDTs include the BioFire Filmarray Respiratory Panel 2.1 and the ePlex Respiratory Pathogen Panel [6].

Vaccines

Messenger ribonucleic acid (mRNA) vaccines, a recent advancement in vaccine technology, have been used to combat COVID-19. These vaccines function by instructing cells to produce a tiny amount of harmless viral genetic material that stimulates an immune response. The mRNA vaccines from Moderna and Pfizer have the ability of COVID-19 vaccines to prevent COVID-19 infections, which has been demonstrated to be very high. Viral vector vaccines deliver the genetic material from the pathogen to the body's cells using a non-lethal virus [7].

Small particles are used in nanoparticle-based immunizations to deliver the vaccine's active components to the body's cells. Since the shape of the virus is mimicked by these particles, the immune system is better able to identify and combat the virus. The Novavax and CureVac vaccines, among others, are nanoparticle-based COVID-19 vaccines. Personalized vaccines are a new area of research that could revolutionize vaccine development. Because these vaccines are made specifically for each individual, they may be more effective and cause fewer side effects. While personalized vaccines are still in the early stages of development, they could be an important tool in the fight against pandemics in the future. To boost the immune response, adjuvants are chemicals that are added to vaccines. They function by encouraging immune system cells to more readily identify and react to the vaccine. Flu vaccines frequently contain adjuvants, and some COVID-19 vaccines do as well.

Antiviral Therapies

Biomedical science has also contributed to the development of antiviral therapies for pandemic prevention and control. They use small-molecule drugs, monoclonal antibodies, RNA-based therapies, CRISPR-based therapies, and broad-spectrum antivirals. Small-molecule drugs are traditional pharmaceuticals that work by binding to specific viral proteins and preventing the virus from replicating. Some examples of small-molecule drugs include remdesivir, which is used to treat COVID-19, and Tamiflu, which is used to treat the flu. Laboratory-produced proteins called monoclonal antibodies imitate the immune system's capacity to combat infections. These treatments function by neutralizing the virus by concentrating on particular viral proteins [8].

The monoclonal antibody cocktail from Regeneron is one of the monoclonal antibody treatments that have been approved for use in COVID-19 emergencies. Targeting the virus' genetic material and limiting its replication are the principles behind RNA-based treatments. Lipid nanoparticles or other delivery systems can be used to deliver these treatments directly to the cells. For the treatment of COVID-19, some RNA-based treatments have produced encouraging outcomes in preliminary clinical trials [9].

The CRISPR-Cas systems, which are gene-editing tools, have recently been adapted for use in antiviral therapies. The CRISPR-based therapies work by targeting specific viral genes and disrupting their function, which can prevent the virus from replicating. While still in the early stages of development, CRISPR-based therapies could offer a new approach to antiviral therapy. Broad-spectrum antivirals are treatments that work well against a variety of viruses. These treatments function by focusing on conserved viral proteins that are present in all viruses. Ribavirin, which is used to treat hepatitis C, and favipiravir, which is used to treat influenza, are two examples of broad-spectrum antivirals [10].

Genome Sequencing

Genome sequencing, which enables researchers to trace the spread of viruses and find novel varieties, has emerged as a crucial tool in pandemic prevention and management. Public health officials can identify and isolate infected people to stop the virus from spreading by using genome sequencing to track how a virus spreads from person to person. The genetic makeup of a virus can be determined through genome sequencing, which can also be used to create vaccinations that target particular proteins or genomic areas. This method was employed in the creation of COVID-19 vaccines, which focus on the virus' spike protein. The genetic makeup of a virus can be determined through genome sequencing, which can also be used to create vaccinations that target particular proteins or genomic areas [11].

The identification of viruses in animals that could be dangerous to human health is another purpose for genome sequencing. This "One Health" strategy can lessen the possibility of pandemics in the future by preventing the spread of zoonotic illnesses. Overall, new uses for genome sequencing in pandemic prevention and control have transformed our knowledge of viral infections and made it possible for researchers to create more potent treatments and vaccines. Genome sequencing is likely to become an even more potent weapon in the fight against pandemics as technology advances [12].

Pandemic prevention and control are rapidly improving thanks to artificial intelligence (AI), which enables researchers to examine massive amounts of data and forecast the spread of viruses. Here are some recently developed biomedical applications of artificial intelligence for pandemic control and prevention.

Artificial Intelligence

Artificial intelligence may be used to simulate the propagation of viruses and forecast how an outbreak will likely develop. Effective resource allocation and the development of focused treatments are both possible with the help of this information. It helps researchers develop their applications related to pandemic situations. To improve viral disease detection and treatment, AI systems can examine patient data and medical imaging. Artificial intelligence can be used, for instance, to spot patterns in chest X-rays that might point to a COVID-19 infection [13].

Artificial intelligence can be used to find prospective medication candidates and forecast how they would interact with viral proteins. This can help find new treatments for viral illnesses and hasten the drug discovery process. It can be used to identify the most promising vaccine candidates and predict how they may interact with the immune system. This information can be used to develop more effective vaccines and improve vaccination strategies. The emerging uses of biomedical science in AI for pandemic control and prevention could fundamentally alter how we recognize, identify, and treat viral diseases. Artificial intelligence will likely become an even more potent weapon in the fight against pandemics as technology advances [14].

Personal Protective Equipment

Personal protective equipment, which gives healthcare professionals and other essential personnel the ability to protect themselves against viral infections, has become an essential tool in pandemic prevention and control. The development of materials PPE is resistant to viruses and bacteria; these materials can increase or decrease PPE's effectiveness.

New materials that can be used in PPE, such as fabrics that risk transmission, are being developed by biomedical scientists. Biomedical researchers are also creating PPE with improved fit and comfort which are easier to wear and more comfortable, like masks that don't irritate the skin or cause glasses to fog up. This can lower the risk of transmission and increase compliance with PPE requirements. Self-cleaning materials, like masks that are coated in a virus-killing substance, are being developed. This can increase PPE's effectiveness, lessen the need for frequent replacements, and have advanced sensors to monitor vital signs and spot early signs of infection. Biomedical researchers are creating sensors that can be incorporated into PPE. By doing this, viral diseases can be treated more effectively and earlier. They are also creating PPE that can be modified to meet specific needs, like masks that are made to fit the wearer's face. This can lessen the risk of transmission and increase the efficiency of PPE [15].

Nanotechnology

Biomedical science and nanotechnology are two fields that have a lot of potential for cross-disciplinary collaboration and innovation. Innovative strategies for COVID-19 prevention and control have been developed as a result of the fusion of biomedical science and nanotechnology.

Some examples of how these two fields have worked together to combat pandemics such as COVID-19 are as follows: With the help of nanotechnology, SARS-CoV-2 is being fought through infection prevention, detection, and treatment. Nanotechnology is a powerful tool with the potential to lower infections because it plays a significant role in the prevention, diagnostics, and therapeutic strategies for COVID-19 management. Among these strategies are the development of antiviral therapeutics or vaccines to introduce antiviral agents into the human body, diagnostic instruments for prompt, accurate, and focused diagnosis, and preventive measures and disinfectants based on nanomaterials. Nanomaterials, like metal nanoparticles, have a high surface-to-volume ratio because they are frequently smaller than one micrometer. Scientists are looking into developing vaccines using nanomaterials. Masks frequently contain nanomaterials like nanofibres and nanofibre webs to reduce the dispersion of large respiratory droplets and shield staff from patient-transmitted droplet transmission [16].

High-performance filtering masks (filtering face piece 2 (FFP2), FFP3, and N95) use a filtering face piece that combines an electrostatic charge and a web of polypropylene microfibers. By using filter materials like nanofibres and nanofibre webs and treating the filter surfaces with antimicrobial substances, the effectiveness of masks in fighting viruses and other microbes has been improved. The hydrophilic biocide film and partially gelled submicron polypropylene nanofibres used in the nanofibre filtering facepiece respirators (FFPR) effectively rendered pathogens inactive. Nanomaterials have also been used to create medical gloves for COVID-19 protection in addition to the mask. Due to the antibacterial properties of silver nanoparticles, for instance, some gloves have been created. It has been demonstrated that the veridical activity of silver nanoparticles (AgNPs) exists. Angiotensin-converting enzyme 2 (ACE2) receptors allow COVID-19 viruses to enter cells, so lowering ACE2 levels could help cut down on the number of infections. It was suggested that using nanotechnology in the gloves to catch the viruses before they enter the cells would be very beneficial [4].

Numerous opportunities exist for improving practicality and ensuring sanitation, thanks to nanotechnology. These nanomaterials include engineered water nanostructures and metallic nanoparticles, primarily titanium dioxide (TiO2) and AgNPs, which have antiviral properties and aid in COVID-19 protection. Deionized water, electrolyzed water, and hydrogen peroxide solutions for microbe inactivation are some of the active ingredients used to make engineered water nano-disinfectants. These nanosanitizers' effectiveness at eliminating microbes from surfaces and the environment was tested. Nanoparticles have drawn a lot of interest in the development of vaccines because of their simple functionalization, controlled-release properties, photothermal and magnetic properties, and tunable size.

They are now widely used by researchers to deliver targeted vaccines to immune cells like dendritic cells (DCs). Selective targeting of DCs with nanomaterials has been achieved using a variety of techniques, demonstrating great promise for the development of low-dose vaccines [17].

Similar to this, nucleic acids were delivered to the target cell using lipid-based nanoparticles. It enables the production of vital viral proteins for immunization or the inactivation of vital viral target genes. By inserting a plasmid containing a DNA sequence encoding the antigen(s) into the appropriate tissues, one vaccination technique has been developed. These vaccines have a problem with insufficient cell delivery. Nucleic acid-based therapeutic products have been administered using cationic lipid nanoparticles to address this problem [16].

The potential for biomedical applications of protein nanoparticles is great. The majority of them are obtained using recombinant technologies. Researchers created self-assembling protein nanoparticles (SAPNs) by oligomerizing monomeric proteins. It was demonstrated that SAPNs could be modified to produce a diameter resembling that of viruses, and as a result, they are recognized as a potential candidate for a vaccine against respiratory viruses. One of the most widely used nanomaterials for quick diagnostics is gold nanoparticles (AuNPs). For instance, in one study, gold nanoparticles were used to identify target viruses' double-stranded DNA (dsDNA). And nanomaterials based on carbon have been widely used to create platforms for COVID-19 diagnostics. Using carbon-based nanomaterials as COVID-19 antiviral therapeutics is possible [18].

Nanozymes are synthetic enzymes made of nanomaterials that function just like natural enzymes do. Because of their exceptional catalytic abilities, quick response times, and capacity for self-assembly, nanozymes are frequently used in the diagnosis and treatment of diseases. Antiviral therapy has a new window of opportunity thanks to nanotechnology. Nanoparticles are adaptable vectors for the targeted delivery of specific therapeutic drugs and viruses due to their flexibility. To improve drug bioavailability, drug delivery, and targeted antiviral activity, organic nanoparticles have been used to deliver antiviral medications like acyclovir, zidovudine, efavirenz, and dapivirine. Exosome delivery to the target cell for therapeutic purposes has recently attracted a lot of attention. They have been introduced as potential biological nanocarriers for COVID-19 treatment in several clinical applications. Coronavirus disease (COVID-19) has also been treated with peptide inhibitors [19].

Overall, the relationship between biomedical science and nanotechnology has led to the development of innovative solutions for pandemic prevention and control. These emerging applications have the potential to not only combat the current pandemic but also be useful for future disease outbreaks. However, it is important to continue to evaluate their safety and effectiveness through rigorous testing and research.

Reviewing emerging applications in depth demonstrates a thorough comprehension of the subject, offering a comprehensive viewpoint. Readers can quickly understand the crucial contributions of biomedical science to pandemic prevention and control by highlighting the main findings. Recognizing various applications, including vaccine development, diagnostics, therapeutics, genomic surveillance, digital health technologies, AI, and PPE innovations, demonstrates a broad understanding of the multifaceted nature of biomedical science. The trustworthiness and dependability of the sources cited have a significant impact on a review's quality. The effectiveness and applicability of the studies and other information sources cited in the review must be critically assessed. Inconsistent results or alternative viewpoints may be ignored if the review heavily relies on a particular subset of studies or sources. To avoid such biases, it is critical to take into account a variety of sources. New discoveries and advancements frequently happen in the field of biomedical science, which is rapidly evolving. The review might not accurately reflect the state of the field today if it doesn't take into account the most recent data or contains incomplete data.

Governments, funding organizations, and research organizations should give biomedical research and development top priority. To effectively prevent and control pandemics, new technologies, treatments, and strategies must be supported. Accelerated research and development of vaccines for newly emerging infectious diseases are necessary. To ensure equitable access globally, this entails funding research on cutting-edge vaccine platforms, enhancing manufacturing capabilities, and setting up strong distribution networks. Priority should be given to the creation of quick and reliable diagnostic tools. Campaigns for public health should emphasize spreading knowledge about the value of immunization, good personal hygiene habits, and adherence to regulations. Disinformation can be fought, and the adoption of preventative measures within communities can be encouraged with clear and accurate communication.

Governments should increase funding for biomedical research and development pertaining to pandemic prevention and control. Supporting the creation of vaccines, treatments, diagnostics, and other cutting-edge solutions falls under this category. To increase preparedness, funding for fundamental studies on emerging pathogens should be allocated. Global problems like pandemics necessitate international cooperation. Governments, research institutions, and industry stakeholders should prioritize collaboration and information sharing. Coordination, information sharing, and the fair distribution of resources can be facilitated by organizations like the WHO and initiatives by international partnerships. Governments must improve their infectious disease surveillance systems. To identify outbreaks early and track their spread, this includes making investments in cutting-edge data collection, analysis, and modeling tools. To enable quick response and containment measures, policymakers should establish efficient early warning systems. Improve risk communication, public health readiness, socioeconomic impacts, public-private partnerships, and healthcare infrastructure. These suggestions highlight important policy areas that can assist governments and stakeholders in better preventing, controlling, and responding to pandemics.

Scientists have innovated new applications using modern technology. Among these experiments, robotic technologies have been extensively used during the current pandemic, including remote patient-doctor communication, ultraviolet (UV) surface disinfection, delivery of vital medical supplies, monitoring vital signs to remind people to practice infection prevention techniques like social seclusion, and scaling up the production of diagnostic tools, medications, and vaccines. The COVID-19 symptom-detection drones and stationary cameras are being deployed by Dragonfly, a drone technology company based in Canada. Drone engineers and enthusiasts use them to deliver vital medical supplies to far-flung hospitals and clinics, as well as to monitor social segregation tactics in large gatherings. Engineers working on new product development techniques are experimenting with ultraviolet light in the so-called UV-C range (100-280 nm). Applications include cleaning the intensive care unit (ICU) and patient rooms, sanitizing technology like smartphones, and possibly reusing N95 masks [20].

This method disinfects by exposing the affected area to UV light rather than using liquid disinfectant. According to studies, UV light can eliminate 99.9% of bacteria, fungi, and viruses when used at the right intensity and duration. The sensor needs to be wearable in order to effectively monitor patient vital signs. As long as it doesn't limit the patient's mobility and can be worn comfortably for extended periods of time, it can be a wristband, a necklace, a body attachment, or even headgear. Numerous technologies, including new fluorogenic aptamers, nanotechnology, quantum dots, advanced image sensors, electrochemical sensors, microfluidics, and others, are being used to improve diagnostic tools. The following is a list of approved diagnostic tools for COVID-19 detection. The majority of these devices use specialized molecular point-of-care test platforms consisting of a reader, a microfluidic cartridge, and a virus detection assay. Modern computing power, novel materials, advanced manufacturing, rapid prototyping, robotics, and cutting-edge tools and techniques enable medical technology (MedTech) innovators to offer much faster, more efficient, safer, and more effective treatments for medical conditions now and in the future.

When a viral disease emerges, future vaccine research needs technology that can be quickly applied to clinical and preclinical experiments with straightforward production. Major problems with recombinant protein subunits, mRNA, and DNA vaccines that have the potential for clinical application are being addressed by nanoengineering. These problems include poor immunogenicity and insufficient induction of inappropriate immune responses. In order to maximize immune activity and protect against proteolysis in the physiological environment, nanoparticulate vaccines are primarily being developed. By altering the physicochemical characteristics of their constituent parts or by presenting metameric forms conjugated or formulated with immune potentiates, nanoplatforms can be created that are optimally engineered for different molecules and specific antigens. Additionally, it's critical to create systems that use molecules and antigens enclosed in nanoparticles to deliver them to the desired location at the right time. It's also crucial to ensure tightly controlled release kinetics. Therefore, nanoengineered vaccines have great potential to be a flexible platform and provide cutting-edge approaches for the creation of upcoming vaccine generations [21].

Still, a number of technological obstacles prevent in-field clinical applications, necessitating more multidisciplinary research. The significance of implementing health regulations and creating technologies based on fundamental and applied sciences is highlighted by the previous and current SARS-CoV outbreaks. All of the topics covered in detail in this review are very helpful for biomedical science and engineering research to combat the current COVID-19 pandemic and any upcoming outbreaks, not only from an experimental point of view but also from methods relying on computational simulation, artificial intelligence, and smart devices. Furthermore, future perspectives in biomedical science and nanomedicine applied to viral diseases are anticipated to primarily focus on early, portable, highly sensitive, and affordable fabrication of diagnosis kits, avoiding complex infrastructures, sophisticated devices, and skilled professions, and the development of theranostic nanomedicine tools, including the development of biodegradable vectors suitable for current patients' treatments. Synthetic biology and protein engineering are expected to work together to create potent therapeutics alongside computer simulations and AI [2].

It's important to highlight the role of genomic surveillance and digital health technologies. Genomic surveillance involves tracking the genetic sequences of pathogens, like viruses, to understand how they evolve and spread. This information is crucial for developing targeted interventions, monitoring variants of concern, and adapting vaccines and treatments [22]. Digital health technologies, including mobile apps, wearable devices, and telemedicine platforms, have played a pivotal role in monitoring and managing public health during pandemics. These technologies enable remote patient monitoring, contact tracing, symptom tracking, and real-time data collection, enhancing the overall response to outbreaks [23].

Artificial intelligence (AI) has emerged as a powerful tool in pandemic management. Machine learning algorithms can analyze large datasets to identify trends, predict disease spread, and optimize treatment strategies. Artificial intelligence-driven drug discovery and vaccine design have accelerated the development process, allowing researchers to identify potential candidates and optimize them for clinical use more efficiently [24].

Innovations in personal protective equipment have also been critical for safeguarding healthcare workers and the general population. Advanced materials and design improvements have led to more comfortable, effective, and sustainable PPE options. For instance, antimicrobial coatings, improved filtration systems, and reusable designs contribute to better protection and reduced environmental impact [25]. Global collaboration and information sharing have become more important than ever in addressing pandemics. International partnerships and organizations like the WHO facilitate coordination, data exchange, and resource allocation [26].

Lessons learned from the COVID-19 pandemic underscore the need for improved early warning systems, rapid response protocols, and strengthened healthcare infrastructure to ensure timely and effective interventions [27]. The field of biomedical research is rapidly evolving, and ongoing investment in research and development is crucial for pandemic preparedness [28]. Governments and funding organizations should allocate resources to support fundamental research on emerging pathogens, advanced diagnostics, innovative therapeutics, and next-generation vaccine platforms. Furthermore, research efforts should aim to enhance our understanding of host-pathogen interactions, immune responses, and the long-term effects of viral infections [29].

As we move forward, interdisciplinary collaboration will play a pivotal role in addressing pandemics [30]. The integration of diverse fields such as biology, engineering, data science, and social sciences will lead to holistic and effective strategies for prevention, control, and response. By fostering an environment of continuous learning, adaptation, and innovation, we can better equip ourselves to tackle current and future health crises on a global scale [31].

## Conclusions

In conclusion, the fight against COVID-19 has greatly benefited from the new uses of biomedical science in pandemic prevention and control. The ongoing research and development in the field provide hope for the control of other infectious diseases in the future. Collaboration between biomedical science and other sectors is crucial in addressing the current and future challenges posed by pandemics. With continued advancements in biomedical science, we can improve our preparedness for future pandemics and minimize the impact on human health and the global economy. The COVID-19 pandemic has also highlighted the necessity for more biomedical science research and development. The gaps in our understanding of the virus and the development of effective and sustainable pandemic prevention and control strategies need to be addressed. Biomedical scientists must continue to work with public health officials, policymakers, and other stakeholders to address these challenges and ensure a comprehensive and effective response to future pandemics.

## References

[1] Omboni S, Padwal RS, Alessa T (2022). The worldwide impact of telemedicine during COVID-19: current evidence and recommendations for the future. Connect Health.

[2] Zamora-Ledezma C, C DF, Medina E, Sinche F, Santiago Vispo N, Dahoumane SA, Alexis F (2020). Biomedical science to tackle the COVID-19 pandemic: current status and future perspectives. Molecules.

[3] McNeill VF (2022). Airborne transmission of SARS-CoV-2: evidence and implications for engineering controls. Annu Rev Chem Biomol Eng.

[4] Ayan S, Aranci-Ciftci K, Ciftci F, Ustundag CB (2022). Nanotechnology and COVID-19: prevention, diagnosis, vaccine, and treatment strategies. Front Mater.

[5] (2023). Use of SARS-CoV-2 antigen-detection rapid diagnostic tests for COVID-19 self-testing. https://www.who.int/publications-detail-redirect/WHO-2019-nCoV-Ag-RDTs-Self_testing-2022.1..

[6] Javalkote VS, Kancharla N, Bhadra B (2022). CRISPR-based assays for rapid detection of SARS-CoV-2. Methods.

[7] Pardi N, Weissman D (2020). Development of vaccines and antivirals for combating viral pandemics. Nat Biomed Eng.

[8] Kumari M, Lu RM, Li MC (2022). A critical overview of current progress for COVID-19: development of vaccines, antiviral drugs, and therapeutic antibodies. J Biomed Sci.

[9] Kim YK (2022). RNA therapy: rich history, various applications and unlimited future prospects. Exp Mol Med.

[10] Rajan A, Shrivastava S, Janhawi Janhawi, Kumar A, Singh AK, Arora PK (2022). CRISPR-Cas system: from diagnostic tool to potential antiviral treatment. Appl Microbiol Biotechnol.

[11] Eyre DW (2022). Infection prevention and control insights from a decade of pathogen whole-genome sequencing. J Hosp Infect.

[12] Quer J, Colomer-Castell S, Campos C (2022). Next-generation sequencing for confronting virus pandemics. Viruses.

[13] Malik YS, Sircar S, Bhat S (2021). How artificial intelligence may help the Covid-19 pandemic: pitfalls and lessons for the future. Rev Med Virol.

[14] Chang Z, Zhan Z, Zhao Z (2021). Application of artificial intelligence in COVID-19 medical area: a systematic review. J Thorac Dis.

[15] Griswold DP, Gempeler A, Kolias A, Hutchinson PJ, Rubiano AM (2021). Personal protective equipment for reducing the risk of COVID-19 infection among health care workers involved in emergency trauma surgery during the pandemic: an umbrella review. J Trauma Acute Care Surg.

[16] Adrah FA, Denu MK, Buadu MAE (2023). Nanotechnology applications in healthcare with emphasis on sustainable covid-19 management. J Nanotechnol Res.

[17] Apostolopoulos V, Thalhammer T, Tzakos AG, Stojanovska L (2013). Targeting antigens to dendritic cell receptors for vaccine development. J Drug Deliv.

[18] López-Sagaseta J, Malito E, Rappuoli R, Bottomley MJ (2016). Self-assembling protein nanoparticles in the design of vaccines. Comput Struct Biotechnol J.

[19] Kumawat M, Umapathi A, Lichtfouse E, Daima HK (2021). Nanozymes to fight the COVID-19 and future pandemics. Environ Chem Lett.

[20] Harris M, Bhatti Y, Buckley J, Sharma D (2020). Fast and frugal innovations in response to the COVID-19 pandemic. Nat Med.

[21] Lim JW, Ahn YR, Park G, Kim HO, Haam S (2021). Application of nanomaterials as an advanced strategy for the diagnosis, prevention, and treatment of viral diseases. Pharmaceutics.

[22] Chintagunta AD, M SK, Nalluru S, N S SK (2023). Nanotechnology: an emerging approach to combat COVID-19. Emergent Mater.

[23] Tilahun B, Gashu KD, Mekonnen ZA, Endehabtu BF, Angaw DA (2023). Mapping the role of digital health technologies in prevention and control of COVID-19 pandemic: review of the literature. Yearb Med Inform.

[24] Alghamdi NS, Alghamdi SM (2022). The role of digital technology in curbing COVID-19. Int J Environ Res Public Health.

[25] Karim N, Afroj S, Lloyd K (2023). Sustainable personal protective clothing for healthcare applications: a review. ACS Nano.

[26] Fanning JP, Murthy S, Obonyo NG, Baillie JK, Webb S, Dalton HJ, Fraser JF (2021). Global infectious disease research collaborations in crises: building capacity and inclusivity through cooperation. Global Health.

[27] (2023). First lessons from government evaluations of COVID-19 responses: a synthesis. https://www.oecd.org/coronavirus/policy-responses/first-lessons-from-government-evaluations-of-covid-19-responses-a-synthesis-483507d6/..

[28] Haldane V, De Foo C, Abdalla SM (2021). Health systems resilience in managing the COVID-19 pandemic: lessons from 28 countries. Nat Med.

[29] Patrick L. Osewe (2023). Pandemic preparedness and response strategies: COVID-19 lessons from the republic of Korea, Thailand, and Viet Nam. https://www.adb.org/publications/pandemic-preparedness-covid-19-lessons.

[30] Madhav N, Oppenheim B, Gallivan M, Mulembakani P, Rubin E, Wolfe N (2023). Pandemics: risks, impacts, and mitigation. Disease Control Priorities, Third Edition (Volume 9): Improving Health and Reducing Poverty.

[31] Zhao Y, Liu L, Zhang C (2023). Is coronavirus-related research becoming more interdisciplinary? A perspective of co-occurrence analysis and diversity measure of scientific articles. Technol Forecast Soc Change.

